# Sleep Disturbances and Behavioral Problems in Children and Adolescents with Autism Spectrum Disorder—A Systematic Review

**DOI:** 10.3390/clinpract15110201

**Published:** 2025-10-30

**Authors:** Cristina Tecar, Lacramioara Eliza Chiperi, Bianca Elena Iftimie, Livia Livint Popa, Valentina Sas, Emanuel Stefanescu, Vitalie Vacaras, Dafin Fior Muresanu

**Affiliations:** 1 RoNeuro Institute for Neurological Research and Diagnostic, 400364 Cluj-Napoca, Romania; cristina.pantelemon@umfcluj.ro (C.T.); livia.popa@umfcluj.ro (L.L.P.); emanuel.stefanescu@brainscience.ro (E.S.); vvacaras@umfcluj.ro (V.V.); dafin.muresanu@umfcluj.ro (D.F.M.); 2 Monza Ares Hospital, 400347 Cluj-Napoca, Romania; 3 Department of Neurosciences, Psychiatry and Pediatric Psychiatry, Iuliu Hațieganu University of Medicine and Pharmacy, 400347 Cluj-Napoca, Romania; biancaiftimie4@gmail.com; 4 Department of Neuroscience, Iuliu Hatieganu University of Medicine and Pharmacy, 400083 Cluj-Napoca, Romania; 5 Third Pediatric Discipline, Faculty of Medicine, Iuliu Hațieganu University of Medicine and Pharmacy, 400124 Cluj-Napoca, Romania; sas.valentina@umfcluj.ro

**Keywords:** autism spectrum disorder, children, sleep disturbances, behavioral problems, aggression, emotional dysregulation, systematic review

## Abstract

**Background/Objectives:** Sleep disturbances are among the most prevalent and persistent comorbidities in children and adolescents with autism spectrum disorder (ASD), affecting up to 83% of this population. These disturbances not only impact the quality of life but are increasingly recognized as significant contributors to behavioral dysregulation. **Methods**: This systematic review synthesizes evidence from 26 studies published between 2010 and 2024, examining the association between sleep problems and behavioral outcomes in individuals with ASD aged 2 to 18 years. **Results**: The findings reveal consistent associations between sleep-onset insomnia, night walking, bedtime resistance, and various behavioral difficulties, including aggression, hyperactivity, and emotional dysregulation. Internalizing symptoms and exacerbation of core ASD features were also linked to chronic sleep problems. Studies employing objective sleep measures, such as actigraphy and polysomnography, further supported these associations by identifying disruptions in sleep architecture correlated with behavioral severity. While most included studies were of moderate to high methodological quality, the limited number of randomized controlled trials and heterogeneity of sleep and behavior assessment tools highlight the need for standardization. **Conclusions**: Overall, the review emphasizes the importance of routine sleep evaluation in ASD clinical care and supports targeted sleep interventions as a potential strategy to reduce behavioral problems and improve developmental outcomes.

## 1. Introduction

Autism spectrum disorder (ASD) is a neurodevelopmental condition characterized by persistent deficits in social interaction and communication in addition to repetitive patterns of interests, activities, or behaviors, as conceptualized in the International Classification of Diseases, Eleventh Revision (ICD-11), and similarly in the Diagnostic and Statistical Manual of Mental Disorders, Fifth Edition (DSM-5) [1]. These core features can range in severity, from poorly integrated verbal and nonverbal communication to a total lack of facial expressions, and from an abnormal social approach to failure to failure to respond to social interactions [1]. The prevalence of ASD has significantly increased over the past decades [2], with current estimates indicating a rate ranging from 1 in 59 to 1 in 40 in the United States [3] and around 1 in 89 children in Europe [4].

Alongside its core symptoms, ASD presents a high rate of medical and psychiatric comorbidities, including epilepsy, intellectual disability, attention-deficit/hyperactivity disorder (ADHD), anxiety, mood disorders, self-injurious behavior, aggression, and sleep disorders [2,5,6]. Among these co-occurring difficulties, sleep disturbances hold particular significance, as they affect approximately 40–83% of children and adolescents with ASD [7], compared to 25–40% in the neurotypical population [3,7,8]. Moreover, although in neurotypical children sleep problems decrease with age, they frequently persist into adolescence for children with ASD [9]. While children experience bedtime resistance, frequent awakenings, sleep anxiety, and parasomnias (especially enuresis, disoriented walking, night terrors, rhythmic movement disorders) [2,10,11], adolescents grapple with sleep onset insomnia, reduced sleep duration, and daytime sleepiness [3,12].

Disrupted sleep has a negative impact on both the affected individual and the caregiver [6,7,8]. It is associated with higher rates of behavioral disturbances [6] (including irritability, hyperactivity, inattentiveness, aggression), as well as the increase in internalizing behavior (anxiety, emotional problems, self-injurious behavior) and ASD core symptoms (repetitive behaviors, difficulties in social reciprocity) [13]. The exacerbation of behavioral and emotional challenges can adversely affect daytime functioning, academic achievement, and social interactions [13], thereby worsening disease prognosis. Furthermore, chronic sleep problems increase parental stress while affecting the parent’s own sleep and functionality [3,6]. These issues can create an economic and societal burden by requiring additional healthcare resources and support services, as well as affecting the broader family’s productivity and well-being.

It has been hypothesized that neurobiological and genetic factors that shape sleep architecture are involved in the etiology of sleep problems in ASD. Polysomnography (PSG) studies identified atypical brain wave organization and maturation in children with ASD [10,14,15], suggesting underlying differences in neural development [14]. The circadian rhythms typically consolidate around 3–4 months of age [12,14]. Disruptions in circadian rhythm regulation, including alterations in clock genes and melatonin production, have been observed in ASD [12,14]. Disruptions in this early circadian establishment can adversely shape subsequent sleep–wake cycles and developmental outcomes [12], potentially intensifying core ASD symptoms.

The application of sleep questionnaires to both carers and individuals with ASD revealed behavioral and environmental factors that further disrupt the sleep–wake cycle. Core ASD symptoms (e.g., restricted interests, heightened arousal, sensory dysregulation, difficulties in self-soothing and adapting to environmental cues) favor heightened stress responses to bedtime changes, eliciting both internalizing and externalizing maladaptive behavior that delays sleep onset [11]. Environmental factors (e.g., excessive screen use, dependency on a specific stimulation, person, object, or setting) additionally impede bedtime routines [14]. Comorbidities, especially ADHD, anxiety, additionally disrupt the sleep–wake cycle [5,13,14].

These factors shed light on how behavioral challenges often observed in children with ASD, arising both from intrinsic and environmental mechanisms, contribute to bedtime resistance and sleep disruption, which in turn exacerbate behavioral dysregulation.

While behavioral problems are frequently addressed in the healthcare evaluation, sleep problems used to be treated as secondary issues. Converging evidence now suggests a reciprocal relationship between poor sleep, behavioral issues [16], and the presentation of core ASD symptoms. Inadequate sleep is tied to poor neuroplasticity and disrupted neural organization, which are essential for attention, memory consolidation, emotional and behavioral regulation —already areas of concern in ASD [2,10,14].

This systematic review aims to examine the association between sleep disturbances and behavioral problems in children and adolescents with ASD. We outline the neurobiological and environmental mechanisms that may contribute to these disruptions and explore their interaction with core symptoms and comorbidities. Thus, we aim to highlight the need for a standardized diagnostic method alongside an evidence-based treatment guideline.

## 2. Materials and Methods

### 2.1. Protocol and Reporting Standards

This systematic review aimed to investigate the association between sleep disturbances and behavioral problems in children and adolescents diagnosed with ASD. This review was conducted in accordance with the PRISMA 2020 statement. A completed PRISMA checklist is provided in the Supplementary Materials (File S1). The protocol was not registered in PROSPERO or any other public database.

### 2.2. Eligibility Criteria

Inclusion criteria were defined using the PICOS framework as follows: population: children and adolescents (0–18 years) diagnosed with ASD; intervention/exposure: assessment or reporting of sleep disturbances; comparison: not mandatory—both comparative and non-comparative studies were eligible; outcomes: behavioral problems, including externalizing, internalizing, or ASD core symptom exacerbation; study design: observational (cross-sectional, cohort, case–control) and interventional studies (e.g., behavioral or pharmacological interventions).

Studies were excluded if they (1) did not report outcome data related to both sleep and behavior; (2) included adult populations; (3) were editorials, reviews, commentaries, or case reports; (4) lacked full-text availability.

### 2.3. Information Sources and Search Strategy

A comprehensive search was conducted in PubMed, Scopus, Embase, Medline, Web of Science, and Cochrane Library using the following search terms:

(“autism spectrum disorder”[MeSH Terms] OR “autism spectrum disorder”[Title/Abstract] OR ASD[Title/Abstract]) AND (“sleep disturbance*”[Title/Abstract] OR insomnia[Title/Abstract] OR parasomnia*[Title/Abstract]) AND (behavior*[Title/Abstract] OR “maladaptive behavior*” OR aggression OR hyperactivity OR internalizing OR externalizing OR emotional regulation) AND (child*[Title/Abstract] OR adolescent*[Title/Abstract])

An equivalent adapted query was applied to each database. The final search was performed in July 2025.

### 2.4. Study Selection and Data Extraction

Two independent reviewers screened the titles and abstracts of all retrieved records using predefined inclusion and exclusion criteria. Full-text articles were then assessed for eligibility. Any disagreements between reviewers during the study selection process were resolved through discussion and, when necessary, consultation with a third reviewer.

The eligibility of studies for each synthesis was decided by comparing the reported population, intervention/exposure characteristics, and behavioral outcome measures with the predefined inclusion criteria. Studies meeting these criteria were grouped according to the type of sleep disturbance, behavioral domain, and study design to ensure homogeneity within each synthesis group.

Data extraction was performed independently by two reviewers and included the following variables: (1) author, year, country; (2) sample size and age; (3) ASD diagnostic criteria; (4) type and assessment of sleep problems; (5) type and assessment of behavioral problems; (6) key findings and statistical associations.

### 2.5. Data Preparation and Handling of Missing Data

Before synthesis, extracted data were reviewed for completeness and consistency. Where necessary, study results were harmonized to ensure comparability (e.g., standardizing behavioral domain terminology and age ranges). Missing summary statistics were reported as “not available” and were not imputed. No data conversions were performed beyond unit standardization or reclassification of behavioral domains for clarity.

### 2.6. Data Synthesis

A qualitative synthesis of the findings was conducted, summarizing the associations between sleep disturbances and behavioral problems in children and adolescents with ASD. Given the clinical heterogeneity across study populations, sleep assessment methods, and behavioral outcome measures, a meta-analysis was not performed.

For the synthesis and presentation of results, the effect measures varied depending on the study design and instruments used. In studies reporting quantitative associations, outcomes were summarized using a comparative table. Due to the limited number of randomized controlled trials (RCTs) and variability in outcome definitions (e.g., internalizing vs. externalizing problems), pooling of data was not feasible.

Instead, key results were presented in narrative form and structured tables, highlighting patterns of association between specific sleep disturbances (e.g., insomnia, bedtime resistance, parasomnias) and behavioral domains (e.g., aggression, emotional dysregulation, hyperactivity). Sensitivity analysis or subgroup comparisons were not applicable due to the small number of homogeneous studies.

Missing summary statistics were reported as “not available” and were not imputed. Only full-text peer-reviewed articles with extractable data were included in the final synthesis.

### 2.7. Risk of Bias and Quality Assessment

The methodological quality of observational studies was assessed using the Newcastle–Ottawa Scale (NOS), while randomized controlled trials (RCTs) were evaluated using the Cochrane Risk of Bias Tool (RoB 2.0). Studies were rated as low, moderate, or high quality.

### 2.8. Ethical Considerations

As this study was a systematic review of previously published literature, no ethical approval was required. All data sources used were publicly available and appropriately cited. The review was conducted in accordance with the PRISMA 2020 guidelines to ensure transparency, reproducibility, and ethical reporting.

## 3. Results

### 3.1. Study Selection Process

**Figure 1:**
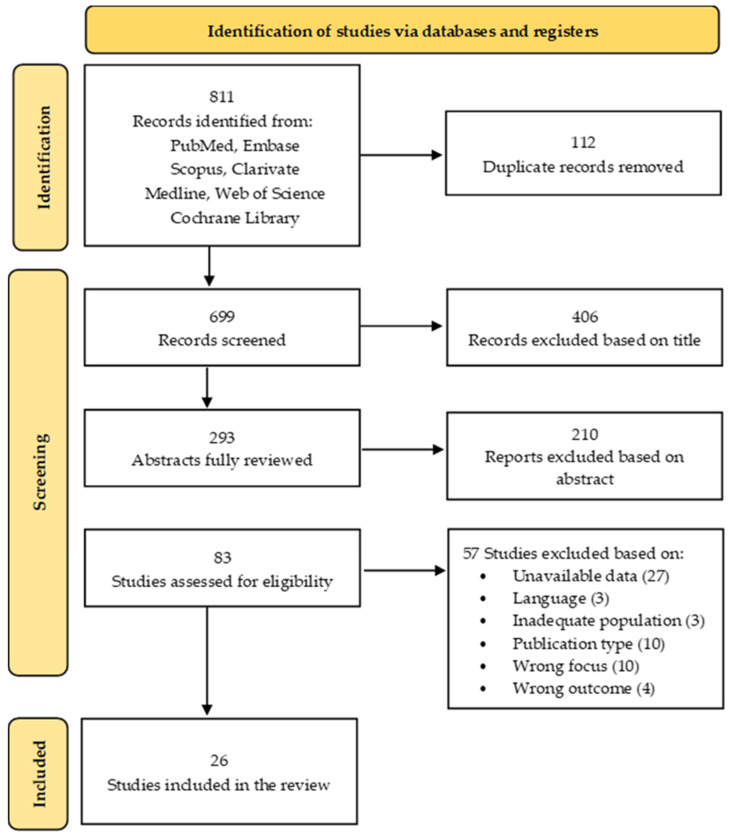
PRISMA flow diagram for new systematic review: selection process of included studies.

A total of 811 studies were initially identified. After removing 113 duplicates, 699 titles were screened. Of these, 293 abstracts were fully reviewed, and 210 were excluded. The full texts of 83 studies were assessed for eligibility, and 26 studies met the inclusion criteria and were included in the final review. The selection process is illustrated in the PRISMA flow diagram shown in Figure 1.

### 3.2. Study Characteristics

A total of 26 studies were included, published between 2010 and 2024, spanning North America, Europe, Asia, and Australia. The studies comprised 20 observational designs (cross-sectional, cohort) and 6 interventional designs (e.g., behavioral or pharmacological interventions). The combined sample included over 3200 children and adolescents with ASD, aged between 2 and 18 years. The characteristics of the studies are reported in Table 1.

### 3.3. Sleep Problems in ASD

Sleep disturbances were widely reported across the included studies, with several recurring patterns emerging. The most common issue identified was sleep-onset insomnia, present in 22 of the reviewed studies, indicating a persistent difficulty in initiating sleep among children and adolescents with ASD. Night walking was the next most frequently reported disturbance, often contributing to fragmented and non-restorative sleep. Bedtime resistance, reflecting oppositional behavior or anxiety related to the bedtime routine, was also frequently noted. In addition, studies described shortened sleep duration and early morning awakenings, which further exacerbated sleep insufficiency. Parasomnias, including sleep terrors and enuresis, were reported less frequently, particularly among younger children or those with co-occurring developmental delays.

To evaluate these disturbances, researchers employed a variety of measurement tools. The Children’s Sleep Habits Questionnaire (CSHQ) was the most commonly utilized instrument, appearing in 17 of the included studies, likely due to its accessibility, standardized structure, and suitability for parent-report. Objective measures such as actigraphy and PSG were used, offering more precise insight into sleep architecture and sleep–wake patterns. Additionally, parent-reported sleep diaries were also employed, providing qualitative context to sleep behaviors observed in home settings.

These findings underscore the heterogeneity and persistence of sleep problems in the ASD population, highlighting the need for comprehensive sleep assessment using both subjective and objective methods.

### 3.4. Behavioral Outcomes

The behavioral outcomes reported across the included studies encompassed a wide range of difficulties, which were generally grouped into three categories: externalizing behaviors, internalizing symptoms, and exacerbation of core ASD features (Table 2). Externalizing behaviors, including hyperactivity, aggression, and irritability, were the most frequently examined. Internalizing symptoms such as anxiety, emotional dysregulation, and depression were investigated; a worsening of core ASD symptoms, including increased stereotypies and reduced social reciprocity, was described in relation to sleep disturbances.

To assess these behavioral domains, various validated instruments were employed. The Child Behavior Checklist (CBCL) was the most commonly used tool, followed by the Strengths and Difficulties Questionnaire (SDQ). Other structured instruments, such as BISCUIT-Part 3, ADOS behavioral metrics, and custom behavioral rating scales, were also applied.

### 3.5. Associations Between Sleep Disturbances and Behavior

A significant association between poor sleep and behavioral disturbances was identified in 80% of included studies. Sleep-onset delay and frequent night walking emerged as the most robust predictors of externalizing behaviors, particularly aggression and hyperactivity. Internalizing symptoms, though less consistently reported, were often linked to chronic insomnia and parasomnias. Importantly, one randomized controlled trial demonstrated that a parent-based telehealth intervention targeting sleep problems not only improved sleep quality but also led to a measurable reduction in behavioral dysregulation, emphasizing the potential of behavioral sleep interventions in this population.

Several studies noted that behavioral problems were not merely secondary to the severity of ASD core symptoms. Instead, behavioral impairments persisted independently, with sleep disturbance scores serving as significant predictors. This was particularly evident in studies employing objective sleep measures such as PSG, where disruptions in REM and NREM sleep architecture were associated with emotional dysregulation and the severity of maladaptive behaviors.

### 3.6. Quality Assessment and Risk of Bias of Included Studies

Overall, the majority of the studies demonstrated moderate to good methodological quality. Most studies clearly reported the use of standardized diagnostic criteria for ASD, such as DSM-based clinical diagnoses. However, a small number of studies did not explicitly specify the diagnostic criteria used, which raises concerns regarding diagnostic consistency.

The assessment of sleep problems was generally well defined across studies, with many employing validated tools such as parent-reported sleep questionnaires, actigraphy, or polysomnography. Similarly, behavioral problems were frequently evaluated using standardized rating scales or structured behavioral assessments. Nevertheless, a few studies lacked detailed reporting on behavioral assessment methodology, limiting the interpretability of their findings.

The methodological quality of the included studies was evaluated using adapted criteria derived from standardized tools commonly employed in systematic reviews, including the NOS, the Joanna Briggs Institute (JBI) checklists, and criteria relevant to RCTs, observational studies, and interventional designs.

#### 3.6.1. Risk of Bias in Randomized Controlled Trials

The risk of bias in RCTs was assessed using the Cochrane Risk of Bias 2.0 (RoB 2) tool, which evaluates five key domains: bias arising from the randomization process, bias due to deviations from intended interventions, bias due to missing outcome data, bias in measurement of the outcome, and bias in selection of the reported result.

Among the included studies, two were identified as RCTs: Ip et al. [18] and Yuge et al. [28]. The study by Ip et al. [18] demonstrated low risk of bias across all domains, indicating strong methodological rigor. In contrast, Yuge et al. [28] showed some concerns regarding the randomization process and the selection of reported results, due to limited details on allocation concealment and prespecification of outcomes (Table 3).

Overall, the RCTs included in this review were of low to moderate risk of bias, supporting the reliability of their findings in relation to sleep interventions in children with ASD.

#### 3.6.2. Risk of Bias in Observational Studies

The methodological quality of the observational studies was assessed using the NOS, which evaluates three key domains: selection (maximum 4 points), comparability (maximum 2 points), and outcome (maximum 3 points). A total score of 7–9 indicates high quality, 5–6 moderate quality, and below 5 indicates low quality.

As shown in Table 4, most studies were rated as moderate to high quality. Studies such as those by Bangerter et al. [31], Reynolds et al. [32], May et al. [34], Sannar et al. [36], Sommers et al. [40], and Tesfaye et al. [41] received the maximum score of 9, reflecting robust selection methods, good control for confounding variables, and strong outcome assessment strategies. Conversely, a subset of studies (e.g., Callahan et al. [17], Hoşoğlu et al. [24]) received lower scores primarily due to limited reporting of selection criteria or lack of comparability between groups. None of the studies were assessed as low quality (score < 5), suggesting an overall acceptable methodological standard across the included observational studies.

## 4. Discussion

### 4.1. Interpretation of the Results

This systematic review summarizes the relationship between sleep disturbances and behavioral problems in children and adolescents with ASD. The findings show that sleep problems are not only highly prevalent among children and adolescents with ASD, but they also point toward a consistent association with behavioral problems across development [3,6].

The high frequency of sleep disturbances in children with ASD may be explained by a combination of neurobiological and behavioral disruptions. Previous studies reported abnormal melatonin production and imbalances in neurotransmitters such as serotonin and GABA. These factors contribute to the disruption of the circadian rhythm, influencing sleep onset, continuity, and quality [6,7]. Objective measurements further support these findings. Polysomnography shows longer sleep latency, reduced efficiency, and REM changes linked to internalizing symptoms [29], while actigraphy highlights fragmented, variable sleep associated with hyperactivity and anxiety [31]. Furthermore, core ASD particularities (heightened arousal, behavioral rigidity, sensory hyper-reactivity) [11] and environmental factors (excessive screen use, dependency on a specific stimulation, person, or setting) likely maintain insomnia features (bedtime resistance, night awakenings) and amplify daytime dysregulation [14].

Among the included studies, more than 80% reported a connection between sleep difficulties and externalizing symptoms such as aggression, hyperactivity, and irritability [23,25,26,27]. Internalizing symptoms (including anxiety, emotional dysregulation, and depression) were also observed, though less consistently across studies [26,40]. Moreover, nine studies reported a worsening of core ASD symptoms, such as impaired social reciprocity and stereotypies [30,36]. These results confirm the growing consensus in the literature that sleep and behavior are closely linked in this population.

While many studies in this systematic review support the association between sleep disturbances and behavioral problems in children with ASD, some findings show variations. One reason for these discrepancies could be the methodological diversity among the included studies. Several relied only on subjective measures, such as caregiver questionnaires (e.g., CSHQ), whereas others included objective measures such as actigraphy or polysomnography [23,29]. In addition, the age of participants varied greatly, from toddlers to adolescents, which might explain differences in behavioral outcomes. Some studies focused on short-term associations, while others focused on longitudinal outcomes, showing that delayed sleep onset in early childhood predicted worse executive functioning later in life [34].

There was also variation in how behavior was measured. Some studies used standard tools like the CBCL or SRS, while others focused on narrower domains such as irritability or social withdrawal [28]. Furthermore, sample sizes varied widely, and studies from different regions may reflect cultural differences in sleep routines or expectations, as seen in the large-scale multicenter survey conducted in China [39].

Despite these differences, some consistent patterns were observed. Sleep disturbances were common in children with ASD, often persisting over time and across developmental stages [34]. Sleep onset delay, bedtime resistance, and night walking were reported in both preschool and school-aged children. Behavioral impairments were significantly associated with sleep disturbances, more strongly in studies that employed objective measures (polysomnography, actigraphy) [29]. Moreover, chronic sleep problems had a negative impact on family life, increasing parental stress and reducing overall quality of life [21,37].

An important contribution of this body of research is the emerging support for interventions. Behavioral approaches (structured bedtime routines, parental training, environmental adjustments) showed promising results in improving both sleep and daytime behavior [21]. Telehealth-delivered interventions also appeared feasible, especially for families in underserved areas [18]. Pharmacological treatments, especially melatonin, were reported as useful for reducing sleep latency, decreasing night awakenings, and increasing total sleep time [28]. However, most studies are short-term and do not address long-term safety.

### 4.2. Limitations

This systematic review has several limitations. The variability in study design, sample size, and measurement tools implies some caution in interpretation. The predominance of cross-sectional designs limits conclusions about causality, while the underuse of objective methods reduces comparability across studies.

### 4.3. Implications for Practice, Policy, and Future Research

The present findings have several practical and policy implications. Clinically, the consistent association between sleep disturbances and behavioral problems in children with ASD underscores the importance of incorporating routine sleep assessment into standard diagnostic and therapeutic protocols. Pediatricians, psychiatrists, and developmental specialists should adopt a multidisciplinary approach that includes screening for sleep difficulties and delivering tailored behavioral sleep interventions, which have shown measurable benefits for both children and families.

From a policy perspective, early identification and management of sleep disturbances could be integrated into community-based autism care pathways and educational support programs. This would reduce the long-term burden of behavioral dysregulation and improve family well-being. Policymakers should support the inclusion of evidence-based sleep management strategies within national autism guidelines and public health frameworks.

For future research, there is a need for longitudinal, multicenter studies using standardized sleep and behavior assessment tools to better define causal relationships and developmental trajectories. Future studies should also explore biological markers (e.g., melatonin metabolism, genetic variants influencing circadian rhythm), as well as the long-term efficacy and safety of pharmacological and non-pharmacological interventions. Establishing shared databases and standardized outcome metrics will improve comparability across studies and enhance evidence synthesis for clinical recommendations.

## 5. Conclusions

This review extends prior work by integrating behavioral, environmental, and biological perspectives on sleep and behavior in children with ASD. Across studies, sleep difficulties were associated with greater severity of internalizing, externalizing, and core ASD symptoms, particularly in those studies using objective sleep measures. Moreover, chronic sleep problems were linked to poorer family quality of life. Our results highlight the importance of including sleep in the broader assessment and support strategies for children with ASD. Interventions should address both behavioral and environmental factors. Tailoring sleep routines to reduce sensory overload, limit screen exposure, and provide consistent pre-sleep cues, while managing comorbid conditions, may improve sleep quality and consequently behavioral regulation. Future research should aim for a consensus on how sleep problems are defined and measured and should prioritize longitudinal studies with larger samples, consistent assessment methods, and a combination of subjective and objective sleep measures. A better understanding of individualized sleep profiles may help tailor interventions and improve both sleep quality and behavioral outcomes.

## Figures and Tables

**Table 1:** Summary table of included studies on sleep and behavioral problems in children with ASD.

Author, Year, Country	Sample Size and Age	ASD Diagnostic Criteria	Sleep Problems: Type and Assessment	Behavioral Problems: Type and Assessment	Key Findings/Statistical Associations
Johnson et al. [9], 2018, USA	Young children	DSM-based clinical diagnosis	Sleep quality; parent report	Disruptive behaviors; parent ratings	Poor sleep linked to increased disruptive behavior
Callahan et al. [17], 2019, USA	Toddlers	Not clearly stated	Sleep problems via parent report	Aggression measured behaviorally	Sleep issues significantly predicted aggression
Ip et al. [18], 2021, Hong Kong	Preschool children	Clinical diagnosis	Insomnia; parent report in RCT	Behavior monitored during intervention	Sleep improved after parent-based intervention
Papadopoulos et al. [19], 2020, Australia	Children with ASD and ID	Clinical diagnosis	Parent-reported sleep problems	Behavior and parent stress measured	Sleep issues correlated with behavior and parent factors
Masi et al. [20], 2021, Australia	Children	Autism spectrum diagnosis	Sleep disorders; parent and clinical assessment	Behavioral attributes (e.g., irritability)	Sleep issues associated with sensory sensitivities
McLay et al. [21], 2021, New Zealand	Children and adolescents	Not specified	Sleep problems; intervention-based	Function-based behavioral assessments	Improved sleep and behavior after intervention
Yavuz-Kodat et al. [22], 2021, France	Children	Clinical ASD diagnosis	Circadian rhythm disturbance; actigraphy	Behavior difficulties via clinical tools	Disrupted rhythms linked with behavioral difficulties
Wang et al. [23], 2022, China	Preschool children	DSM-based criteria	Parent-reported sleep disturbances	Quantified behavior problems	Sleep issues predicted behavioral challenges
Hoşoğlu et al. [24], 2022, Turkey	Newly diagnosed children	Clinical ASD diagnosis	Parent-reported sleep issues	Not detailed	Sleep affected by related child and family factors
Distefano et al. [25], 2021, Italy	Preschoolers	Clinical diagnosis	Sleep disorders; clinical tools	Behavior problems via standardized scales	Sleep and behavior strongly associated
Fadini et al. [26], 2015, Brazil	Children with ASD	Unclear	Sleep disorders via questionnaires	Standardized behavior measures	Sleep impacted multiple behavioral domains
Leader et al. [27], 2021, Ireland	Children and adolescents	Not specified	Sleep, GI, affective symptoms; parent report	Challenging behavior; multiple tools	Sleep was a key predictor of behavioral problems
Yuge et al. [28], 2016, Japan	Children with NDD	Confirmed diagnosis	Long-term melatonin intervention	Aberrant behavior monitoring	Melatonin improved sleep and behavior
Nguyen et al. [29], 2018, USA	Children	Clinical diagnosis	Polysomnography: NREM/REM structure	Neurobehavioral outcomes	Sleep architecture linked to behavior regulation
Lindor et al. [30], 2022, Australia	Children with ASD	Not clearly defined	Parent-reported sleep disturbance	ASD core symptoms + behavior	Sleep and symptom severity predicted problems
Bangerter et al. [31], 2020, USA	Children with ASD	Clinical diagnosis	Actigraphy and parent report	Behavior via multiple raters	Higher sleep variability = worse behavior
Reynolds et al. [32], 2019, USA	ASD + ADHD children	Confirmed diagnoses	Parent-reported sleep behaviors	Externalizing behavior scales	Co-morbidity increased behavioral risk
Favole et al. [33], 2022, Italy	Young children with DD	Developmental delay + ASD	Sleep and emotional dysregulation; parent report	Emotional regulation; behavioral tools	Sleep strongly tied to emotional problems
May et al. [34], 2015, Australia	High-functioning children	Clinical diagnosis	Longitudinal sleep tracking	Behavior over time	Worsening sleep predicted later behavior issues
Hirata et al. [35], 2016, Japan	Preschoolers	Clinical ASD	Parent-reported sleep problems	Problem behaviors via scales	Frequent sleep problems linked to behavior
Sannar et al. [36], 2020, USA	Hospitalized children with ASD	Psychiatric clinical data	Sleep problems; medical records	Maladaptive behaviors	Severe sleep problems tied to behavior severity
Martinez-Cayuelas et al. [37], 2021, Spain	Children and adolescents	Confirmed ASD	Circadian rhythm and sleep assessments	Behavioral difficulties; questionnaires	Sleep and rhythms predicted behavior issues
Estes et al. [38], 2017, USA	School-age children	Clinical diagnosis	Sex differences in sleep disturbances	Behavior outcomes analyzed by sex	Sex-specific sleep-behavior patterns
Chen et al. [39], 2021, China	Children with ASD	Multicenter clinical study	Parent-reported sleep issues	Not detailed	Sleep problems prevalent across centers
Sommers et al. [40], 2022, Australia	Autistic children	Confirmed ASD	Network analysis of sleep data	Emotional and behavioral problems	Sleep was central in behavior regulation
Tesfaye et al. [41], 2021, Canada/UK	Young school-aged children	Parent-reported ASD	Early sleep data; longitudinal	Teacher-reported executive function	Poor early sleep predicted later deficits

Abbreviations: ASD = autism spectrum disorders; DSM = Diagnostic and Statistical Manual of Mental Disorders; ID = Intellectual Disability; GI = gastrointestinal symptoms; NDD = Neurodevelopmental disorders; NREM/REM = Non-Rapid Eye Movement/Rapid Eye Movement; ADHD = Attention-Deficit/Hyperactivity Disorder; DD = Developmental delay.

**Table 2:** Behavioral outcomes in children with ASD.

Internalizing Behaviors	Externalizing Behaviors
Anxiety	Aggression
Depression	Hyperactivity
Emotional problems	Defiance
Withdrawal	Impulsivity

**Table 3:** Risk of bias in included randomized controlled trials.

Study	Bias from Randomization Process	Bias Due to Deviations from Intended Interventions	Bias Due to Missing Outcome Data	Bias in Measurement of the Outcome	Bias in Selection of the Reported Result	Overall Risk of Bias
Ip et al. [18], 2021	Low	Low	Low	Low	Low	Low
Yuge et al. [28], 2016	Some concerns	Low	Low	Low	Some concerns	Some concerns

**Table 4:** Risk of bias in included observational studies.

Study	Selection (0–4)	Comparability (0–2)	Outcome (0–3)	Total Score (0–9)	Overall Quality
Johnson et al. [9]	3	1	2	6	Moderate
Callahan et al. [17]	2	1	2	5	Moderate
Papadopoulos et al. [19]	3	1	2	6	Moderate
Masi et al. [20]	3	1	2	6	Moderate
Yavuz-Kodat et al. [22]	4	1	3	8	High
Wang et al. [23]	4	1	2	7	High
Hoşoğlu et al. [24]	3	0	2	5	Moderate
Distefano et al. [25]	4	1	3	8	High
Fadini et al. [26]	3	1	2	6	Moderate
Leader et al. [27]	3	1	2	6	Moderate
Lindor et al. [30]	3	1	2	6	Moderate
Bangerter et al. [31]	4	2	3	9	High
Reynolds et al. [32]	4	2	3	9	High
Favole et al. [33]	3	1	2	6	Moderate
May et al. [34]	4	2	3	9	High
Hirata et al. [35]	3	1	2	6	Moderate
Sannar et al. [36]	4	2	3	9	High
Martinez-Cayuelas et al. [37]	3	1	2	6	Moderate
Estes et al. [38]	4	2	3	9	High
Chen et al. [39]	4	1	2	7	High
Sommers et al. [40]	4	2	3	9	High
Tesfaye et al. [41]	4	2	3	9	High

## Data Availability

No new data were created or analyzed in this study. Data sharing is not applicable to this article.

## Supplementary Materials

The following supporting information can be downloaded at: https://www.mdpi.com/article/10.3390/clinpract15110201/s1, File S1: PRISMA 2020 checklist. Ref. [42] is cited in Supplementary Materials.

## Abbreviations

The following abbreviations are used in this manuscript:

ASD	autism spectrum disorder
ADHD	attention-deficit/hyperactivity disorder
PSG	polysomnography
RCT	randomized controlled trials
NOS	Newcastle–Ottawa Scale
CSHQ	Children’s Sleep Habits Questionnaire
CBC	Child Behavior Checklist
SDQ	Strengths and Difficulties Questionnaire
